# Assessing the effectiveness of OTC Advertising on artificial tear drops from an experiential marketing perspective

**Published:** 2019

**Authors:** Consuela-Mădălina Gheorghe, Victor Lorin Purcărea, Iuliana Raluca Gheorghe

**Affiliations:** * “Carol Davila” University of Medicine and Pharmacy, Department of Marketing and Medical Technology, Bucharest, Romania

**Keywords:** experiential marketing, pharmaceutical advertising, over the counter drugs, ophthalmology advertising effectiveness, artificial tear drops

## Abstract

**Introduction:** It is acknowledged that leading pharmaceutical companies lately spend more on marketing than they are investing in research and technology development. Romania registers one of the largest market growths in the pharmaceutical industry from Central and Eastern Europe, and it is one of the main investors on the advertising market. The rapid changes in the pharmaceutical landscape have demanded for organizations to re-evaluate their infrastructure and the information delivery methods, as well as cut through the clutter and build competitive advantages by using effective advertising. The dry eye is a commonly disease encountered worldwide, which is treated with the help of over-the-counter (OTC) artificial tear drops.

**Aim:** The aim of this study was twofold: to determine the profile of the Romanian consumer who uses artificial tear drops and to assess the components of experiential marketing used in a TV advertisement, which have the highest influence on the consumer’s perception of effective advertising.

**Material and method:** We selected a TV advertisement that used the magical concept of the artificial tear drops in the shape of water in a desert area, suggesting an eye irritation. The instrument for data collection was a self-administered questionnaire based on the watched advertising spot about the OTC artificial tear drops. The sample was made up of 384 participants and the sampling method was the snowball technique. Moreover, a model using Structural Equation was validated in order to assess the established relationships between the experiential marketing components and the effectiveness of the OTC artificial tear drops advertising.

**Findings:** The findings showed that the demographic profile of the OTC artificial tear drops consumer is a female, with the mean age of 39 years, who graduated from university, with an average income of 2500 RON (Romanian currency), single, and with an office job. The mean number of hours spent in front of a computer per day was 10. The structural equation model revealed that the component think experience has the highest direct influence on the consumer’s perception of an advertisement about OTC artificial tear drops as being effective.

**Discussion:** The pharmaceutical market is different from other markets in that the decision maker is not the purchaser except for the OTC drugs that do not require a receipt from a physician. Think experience focuses on rational decision-making and problem solving but in a creative way.

## Introduction

It is acknowledged that leading drug companies lately spend more on marketing than they are investing in research and technology development [**1**]. Romania registers one of the largest market growths in the pharmaceutical industry from Central and Eastern Europe and has had an over 4% growth in 2018. Moreover, the Romanian pharmaceutical market is expected to grow significantly by 2020 with 23.9%, but this growth compared to other countries is rather insignificant [**2**]. 

Further, the dynamic changes in the pharmaceutical landscape have demanded for organizations to re-evaluate their infrastructure and the information delivery methods. Heavy competition for market share, as well as a variety of other industry driven factors, emphasized the necessity of integrating and using disparate source systems in order to provide a single version of their products, and reach mass population through advertising. Therefore, the utmost concern of specialists is to make advertising effective. In reaching the point of effectiveness, pharmaceutical organizations have to reflect the needs and desires of consumers in the advertisement’s message, to cut through the clutter and gain his attention, by using experiential marketing strategies. 

Around 2016, the pharmaceutical market in Romania was still dominated by the segment of drugs released on prescriptions (Rx), which registered sales of 8.8 billion RON in comparison with 2008 when the sales did not exceed 5 billion RON. However, the sales in this field have risen slowly, different from the whole market, so that, during 2008 and 2016, the market share has dropped below 70%. In 2017, the over-the-counter (OTC) drug segment reached 2.6 mld. RON and had 20.2% of the market share [**3**]. 

The pharmaceutical market is the third investor in TV advertising, after telecommunications and cosmetic products. According to Zenith Romania Advertising Expenditure Forecast Report, in 2014, the pharmaceutical industry was the main investor in advertising in Romania, ranking the first among the industries that buy advertisements, revealing a 13% share of the advertising market share [**4**].

In ophthalmology, the dry eye disease is a prevalent condition worldwide, and it is expected to increase yearly, affecting 33.7% of the population [**5**]. Despite the fact that current strategies related to diagnosing the dry eye rely exclusively on the subjective-reported symptoms and on the objective ocular tests [**6**], the first line of pharmacotherapy treatment consists of over-the-counter (OTC) artificial tear drops [**7**]. 

**The aim of this study was twofold:**

(i) to determine the profile of the Romanian consumer who uses OTC artificial tear drops;

(ii) to assess the components of experiential marketing used in a TV advertisement, which have the highest influence on the consumer’s perception of effective advertising. 

## Background

**• Direct to Consumer Advertising in the pharmaceutical industry**

Direct to Consumer Advertising (DTCA) is a new method of approaching consumer pharmaceutical products by employing TV promotional spots. In Romania, the advertising of drugs is strictly controlled by regulatory organisms such as the National Agency for Medicines and Medical Devices, but advertising directed towards the audience is allowed for over-the-counter drugs. Moreover, the sales of over-the-counter drugs continued to rise, while being stimulated by innovation, promotion, and access through distribution channels. At present, the OTC market is a key source of extending the business and offers competitive advantage. However, in order to advertise their products, the OTC drug suppliers require a mandatory approval for the materials that reach the general audience and represent an extra warranty for the information received by the consumers. Therefore, the warranty lies in the fact that the consumers receive revised information, which has been approved by authorities, is real, and does not mislead them. According to title XVII stipulations, chapter VIII in Law no. 95/ 2006, regarding the Romanian Reform in Health, with further amendments, the National Agency for Medicines and Medical Devices is the authority responsible for the evaluation and the approval of the advertising materials and of any other type of advertising regarding drugs designated for human usage. In 2010, the Scientific Council of the National Agency for Medicines and Medical Devices released “The guide regarding the evaluation of the advertising of the drugs used by humans”. 

The mandatory elements that any type of advertising, aimed at the general audience, should have, are the following [**8**]: 

- name of the drug; 

- information necessary for a correct use of the drug; 

- an explicit and easy to read invitation to carefully read the usage, mentioned either on the inside or the outside of the wrapper.

On one hand, the advantages brought by DTCA are the following: increase awareness about diseases, educate consumers about the treatment options, motivate consumers to contact their physicians and engage in a dialogue about health concerns, increase the likelihood that consumers would receive appropriate care for conditions that are frequently under-diagnosed and under-treated, encourage compliance with prescription drug treatment regimens [**8**], and, on the other hand, the disadvantages of DTCA are the following: distortion of health information, provides only partial truths about conditions and cures, leaves consumers with false perceptions regarding the efficacy of drug treatments, and, advertisements do not fully disclose drug risks and overstate the prevalence of health conditions [**9**].

**• Experiential Marketing Framework**

Experiential marketing is a valuable strategy for pharmaceutical industries, which may be used to target specific consumers. Moreover, experiential marketing delivers a compelling product experience that is appealing to the desires and needs of its consumers, and, it is defined as a memorable experience that goes deeply into the customer’s mind [**10**]. According to Smilansky, experiential marketing is defined as a process of identifying and satisfying customer needs and desires profitably, by engaging them through two-way communications that bring experiences to life and add value to the target audience [**11**].

Relying on the strategic experience model, Schmitt divided the components of experiential marketing into 5 categories [**12**]: 

- Sense Experience Component refers to the experience that consumers gain while using their senses, namely, sight, hearing, touch, smell and taste; 

- Affect Experience Component implies that the experience of the consumer is gained through the stimulation of emotions, moods and feelings when using a product; 

- Think Experience Component refers to the stimulation of the consumer’s creativity at a cognitive level and it engages customers in solving real problems;

- Act Experience Component highlights the consumer’s activities, through which he or she will engage in direct physical contact with the product and whose purpose is to trigger certain behavioral habits and lifestyles; 

- Relate Experience Component represents an experience that allows the consumer to establish ties with various entities and communities through the process of consumption. 

**• Effective advertising from an experiential marketing perspective**

From an experiential marketing perspective, sense and affect experience strategies may be employed by specialists in advertising to trigger the expected outcomes in determining consumer behavioral change [**31**]. 

Further, think experience ensures that the changing behavior occurs by learning process. In the case of a think experience, specialists in advertising implement convergent thinking messages, where a well-defined and rational problem is rigorously solved when using a surprise message and an intriguing component, which should rise curiosity and challenge the consumer. 

Persuasion elements embedded in a Relate experience advertising message establish reinforcement and change in a consumer’s attitude or emotion, using logic and proof. 

The Act experience focuses on actions and behaviors. Hence, behavior is regarded by advertisers as the ideal outcome if consumers try the product, and, at the same time, it suggests that consumers would adopt different patterns of behaviors and lifestyles that take the shape of actions related to the physical body, lifestyles, as well as interactions with other people. 

**• Effective advertising of the dry eye syndrome**

Dry eye is a commonly encountered disorder of the eye’s surface, characterized by the degradation of the fluid layer of the eye, which is known as the tear film, as well as by the increased eye inflammation symptoms such as pain, foreign body sensation, dryness or irritation, burning, light sensitivity, redness, and eyelash debris [**5**,**14**]. 

The etiology of dry eye has two major classifications: tear deficiency and excessive tear evaporation [**15**]. Both treatment strategies of the two classifications have limitations, meaning that in some individuals, little or no correlation has been made between their reported symptoms and ocular surface damage, and there is an extreme variation in the objective test performance in diagnosing the dry eye syndrome [**16**]. However, the first treatment of the dry eye is the OTC artificial tear drops, which may take the shape of drops, gels, ointments, or lubricants [**7**]. The OTC artificial tear drops mimic the layers of the tear film in order to maintain the ocular hydration [**17**]. Specifically, the primary role of the OTC artificial eye drops is to supplement an individual’s tears and to provide the necessary eye lubrication in order to prevent tear evaporation and stabilize the tear film. As such, artificial tears are reported to provide “temporary relief of burning and irritation due to dryness of the eye”, “temporary relief of discomfort due to minor irritations of the eye or to exposure to wind or sun”, or, “to be protectant against further irritation or to relive dryness of the eye” [**5**]. 

In depicting the dry eye symptoms, most advertising TV spots use metaphors such as “sand in the eye”, “spider web like irritation” and “desert like irritation”. 

## Materials and methods 

• Participants. Participants comprised 400 people from Bucharest, who were selected using the snowball technique. Still, the inclusion criteria encompassed individuals who used the computer more than 6 hours per day, wore eyeglasses or contact lens, have been diagnosed with dry eye during the last 6 months before the study, with no self-reported psychiatric morbidity, and no other impairments which would cause difficulty in understanding and completing a questionnaire. 

Out of 400 individuals, 384 agreed to participate (85%) in the present study, and all respondents completed written consent forms. 

• Procedure. The design of the study was cross-sectional. The data collection was conducted between April 2019 and June 2019. The study instruments, namely, the questionnaires were printed and inserted in sealable envelops to ensure confidentiality.

The selection of the TV advertisement consisted of a spot, which focused on the OTC artificial tear drops in the shape of a sprayed mist on the eyelid. The dry eye symptoms were depicted using the metaphor of a desert and the mist was the water in the desert. Moreover, the respondents visualized the 20 seconds spot before completing the questionnaires. 

All respondents received a questionnaire, which comprised of two sections:

- The first section gathered socio-demographic information about the OTC artificial tear drop consumers, such as age, gender (male/ female), education (primary school/ high-school/ university/ other), income reported in RON, marital status (married/ single/ divorced/ other), employment status (office work/ field work), and the number of hours spent in front of a computer per day. 

- The second section consisted of statements that describe the experiential marketing components. The items helped in identifying the component with the highest influence on the OTC artificial tear drop consumers after viewing the TV advertisement. Specifically, we assessed which experiential marketing component elements embedded in the TV advertisement about the OTC tear drops was more effective. 

The perceived effectiveness of the advertisement was measured with 5-point Likert scale, which ranged from 1-Strongly Disagree to 5-Strongly Agree. The statements presenting the experiential marketing components as well as the effective advertising, included in the second section of the questionnaire, are illustrated in **Table 1**.

**Table 1:** The scales that measure the effectiveness of an advertisement about OTC artificial tear drops from an experiential marketing perspective

Variable	Number of items	Description of items
Sense Experience	3	The ad triggered my senses very much.
		The ad was perceptually interesting
		The product design of the artificial tear drops advertised was very attractive.
Affective Experience	6	The ad put me in a certain mood.
		The ad made me respond in an emotional manner.
		The ad made me feel comfortable about using the artificial tear drops.
		The ad made me feel it provided useful information.
		The ad made me feel it is believable.
		The ad made me feel it is secure to use the artificial tear drops.
Act Experience	4	The ad made me think about my lifestyle.
		The ad reminded me of the activities I can do after administering the artificial tear drops.
		Every indication in the ad made it easy for me to understand the features of the artificial tear drops.
		The information about the artificial tear drops included in the ad attracted me to further buy them.
Relate Experience	3	I could relate to the people in the ad through the usage of the artificial tear drops.
		The features of the artificial tear drops provided in the ad made me think of the importance of having it handy.
		The features provided by the artificial tear drops in the ad made me get closer to it.
Think Experience	3	The artificial tear drops’ features intrigued me.
		The properties of the artificial tear drops stimulated my curiosity in using it.
		The ad reminded me of the activities I could do after administering the artificial tear drops.
Effective advertising	6	Effective advertising created awareness about the artificial tear drops.
		Effective informational ad created the interest in the artificial tear drops.
		Effective advertising motivated me to buy the artificial tear drops.
		Effective advertising helped me gain knowledge about the artificial tear drops.
		Associating an ad with somebody helped me remember the artificial tear drops.
		Effective advertising could change my attitudes towards the artificial tear drops.

In order to investigate the effectiveness of the advertisement and to determine the experiential marketing components with the highest impact, a structural equation modeling was conducted in WarpPLS version 6.0 [**18**]. 

**Fig. 1:**
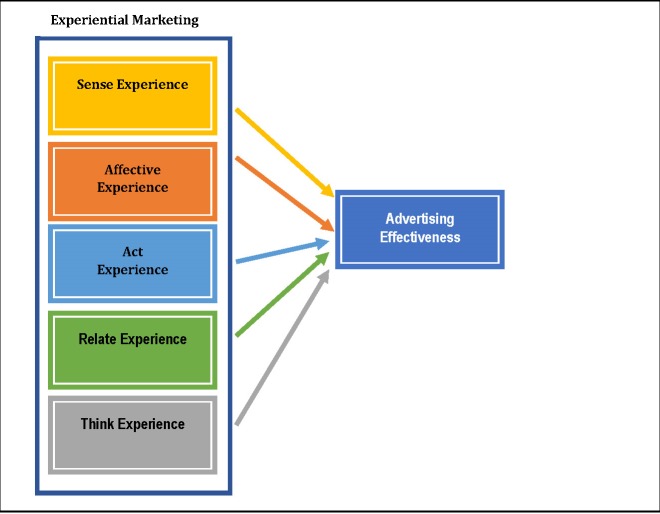
The conceptual framework of the model

Based on the established relationships between the experiential marketing components and the effectiveness of advertising, the following hypotheses were elaborated (**Fig. 1**):

H1: There is a positive relationship between the elements suggesting a sense experience in an advertisement about OTC artificial tear drops and its effectiveness. 

H2: There is a positive relationship between the elements suggesting an affective experience in an advertisement about OTC artificial tear drops and its effectiveness. 

H3: There is a positive relationship between the elements suggesting an act experience in an advertisement about OTC artificial tear drops and its effectiveness. 

H4: There is a positive relationship between the elements suggesting a relate experience in an advertisement about OTC artificial tear drops and its effectiveness. 

H5: There is a positive relationship between the elements suggesting a think experience in an advertisement about OTC artificial tear drops and its effectiveness. 

## Findings

The demographic profile of the Romanian OTC artificial tear drops consumer is a female (n=206), with the mean age of 39 years, who has graduated from university, with an average income of 2500 RON, single, and with an office job. The mean number of hours spent in front of a computer per day was 10. 

**Fig. 2:**
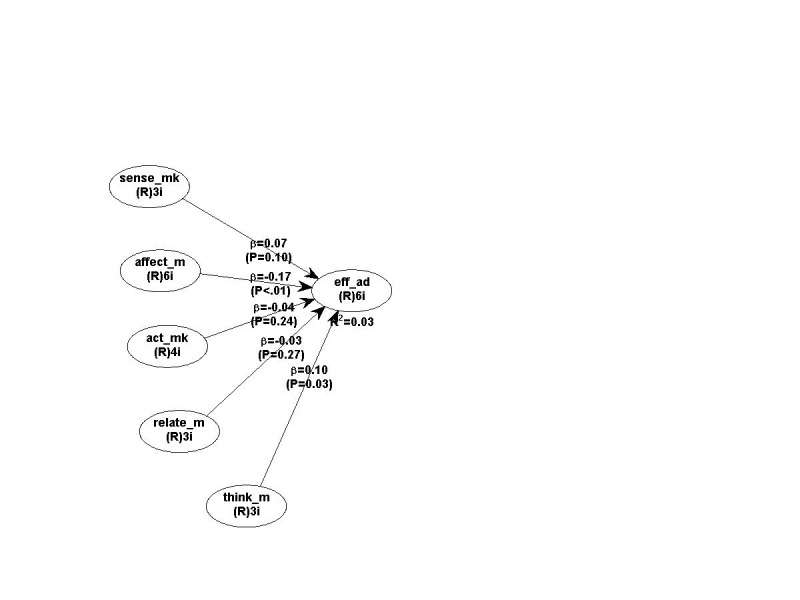
The model’s outcome in WarpPLS

The effectiveness of the TV advertisement about the OTC artificial tear drops was assessed with the help of a model, which included the independent components of experiential marketing and the dependent variable, the effectiveness of the TV advertisement. The validity and the reliability of the model were performed in WarpPls, by using specific goodness of fit indices. As such, for all the experiential marketing components and for the effectiveness of the TV advertisement, Cronbach’s alpha coefficients were above 0.7, meaning that the internal consistency was optimally approached, and the model’s goodness-of-fit was confirmed by the following outcomes: the Average Path Coefficient (APC) value was 0.08 at a p level <0.05, Average R-squared (ARS) had a value of 0.02 at a p level < 0.05, whereas the Average variance inflation factor (AFVIF) had a value of 1.007, which was lower than the threshold of 5 [**18**]. Moreover, the PLS-SEM model, the path coefficients, and their associated p values are illustrated in **Fig. 2** and the hypotheses’ testing is described in **Table 2**. 

**Table 2:** Hypotheses’ testing results

No.	Hypothesis	Beta	P value	Status
H1	There is a positive relationship between the elements suggesting a sense experience in an advertisement about OTC artificial tear drops and its effectiveness.	0.07	p=0.10	Not supported
H2	There is a positive relationship between the elements suggesting an affective experience in an advertisement about OTC artificial tear drops and its effectiveness.	-0.17	p<0.01	Not Supported
H3	There is a positive relationship between the elements suggesting an act experience in an advertisement about OTC artificial tear drops and its effectiveness.	-0.04	p=0.24	Not supported
H4	There is a positive relationship between the elements suggesting a relate experience in an advertisement about OTC artificial tear drops and its effectiveness.	-0.03	p=0.27	Not supported
H5	There is a positive relationship between the elements suggesting a think experience in an advertisement about OTC artificial tear drops and its effectiveness.	0.10	p=0.03	Supported

## Discussion

The pharmaceutical market is different from other markets in that the decision maker is not the purchaser except for the OTC drugs that do not require a receipt from a physician. The DTCA is an “effort” made by pharmaceutical companies to promote their OTC products directly to consumers [**19**]. The most encountered DTCA in Romania is the “reminder advertisement”, which provides the information about a medical condition and includes the product name, the strength, dosage form, and the recommendations of reading the prospect of the drug before administering it. 

The aim of this study was two fold: to determine the profile of the Romanian consumer who uses OTC artificial tear drops and to assess the components of experiential marketing used in a TV advertisement that have the highest influence on the consumer’s perception of effective advertising. 

Findings revealed that the vast majority of respondents who use OTC artificial tear drops are females, with the mean age of 39 years, who have graduated from university, with an average income of 2500 RON, with an office job, and who spend more than 10 hours in front of a computer.

The structural equation model emphasized that the experiential marketing component with the highest direct influence on the consumer’s perception of an advertisement about OTC artificial tear drops as being effective is the think experience. This was no surprise as think experience is based on convergent thinking and points out a more rational approach but in a creative way. From an experiential marketing perspective, the TV advertisement should integrate a surprise element as well as an intriguing message, with the help of technology and innovation in the field [**20**]. 

## Conclusion

The dry eye is a disease commonly encountered worldwide. The impact of dry eye is expected to increase significantly in the next years, and the primary pharmacotherapy recommended in such cases is the artificial eye drops. These ophthalmic substances may be bought without a medical prescription, being assigned to the over-the-counter category (OTC). The vast majority of pharmaceutical companies produce OTC artificial tear drops but in order to cut through the clutter and have a competitive advantage they need to develop and implement effective advertising strategies. One such strategy is to enclose experiential marketing elements in the advertising’s message. In fact, experiential marketing elements refer to five components in the shape of experiences: sense, affective, act, relate and think. 

The findings of this study revealed that OTC artificial tear drop consumers perceive an advertising about OTC artificial tear drops as being effective if think experience elements are embedded in the message. Think experience focuses on rational decision-making and problem solving but in a creative way. 
